# Plasma Amyloid-β, Total Tau, and Neurofilament Light Chain Across the Alzheimer’s Disease Clinical Spectrum: A Population-Based Study

**DOI:** 10.3233/JAD-230932

**Published:** 2023-11-07

**Authors:** Yi Dong, Tingting Hou, Yuanjing Li, Rui Liu, Lin Cong, Keke Liu, Cuicui Liu, Xiaolei Han, Yifei Ren, Shi Tang, Bengt Winblad, Kaj Blennow, Yongxiang Wang, Yifeng Du, Chengxuan Qiu

**Affiliations:** a Department of Neurology, Shandong Provincial Hospital Affiliated to Shandong First Medical University, Jinan, Shandong, P.R. China; b Institute of Brain Science and Brain-inspired Research, Shandong First Medical University & Shandong Academy of Medical Sciences, Jinan, Shandong, P.R. China; c Key Laboratory of Endocrine Glucose & Lipids Metabolism and Brain Aging in Shandong First Medical University, Ministry of Education of the People’s Republic of China, Jinan, Shandong, P.R. China; d Department of Neurology, Shandong Provincial Hospital, Shandong University, Jinan, Shandong, P.R. China; e Aging Research Center and Center for Alzheimer Research, Department of Neurobiology, Care Sciences and Society, Karolinska Institutet-Stockholm University, Stockholm, Sweden; f Department of Neurobiology, Care Sciences and Society, Division of Neurogeriatrics, Center for Alzheimer Research, Karolinska Institutet, Stockholm, Sweden; g Theme Inflammation and Aging, Karolinska University Hospital, Huddinge, Sweden; h Department of Psychiatry and Neurochemistry, Institute of Neuroscience and Physiology, The Sahlgrenska Academy at the University of Gothenburg, Mölndal, Sweden; i Clinical Neurochemistry Laboratory, Sahlgrenska University Hospital, Mölndal, Sweden

**Keywords:** Alzheimer’s disease, diagnostic accuracy, mild cognitive impairment, plasma biomarkers, population-based study

## Abstract

**Background:**

Plasma biomarkers have emerged as a promising approach for characterizing pathophysiology in mild cognitive impairment (MCI) and Alzheimer’s disease (AD).

**Objective:**

We aimed to characterize plasma biomarkers for AD and neurodegeneration across the AD clinical continuum, and to assess their ability to differentiate between AD, MCI, and normal cognition.

**Methods:**

This population-based study engaged 1,446 rural-dwelling older adults (age ≥60 years, 61.0% women) derived from MIND-China; of these, 402 were defined with MCI and 142 with AD. Plasma amyloid-β (Aβ), total tau (t-tau), and neurofilament light chain (NfL) concentrations were analyzed using the Simoa platform. Data were analyzed using linear and logistic regression models, and receiver operating characteristic (ROC) analysis.

**Results:**

Across the AD clinical spectrum, plasma Aβ_40_ and NfL increased, whereas Aβ_42_/Aβ_40_ ratio decreased. Plasma t-tau was higher in people with AD dementia than those with MCI or normal cognition. Plasma NfL outperformed other biomarkers in differentiating AD from normal cognition (area under the ROC curve [AUC] = 0.75), but all plasma biomarkers performed poorly to distinguish MCI from normal cognition (AUC <0.60). Plasma NfL in combination with age, sex, education, and *APOE* genotype yielded the AUC of 0.87 for differentiating between AD and normal cognition, 0.79 between AD and MCI, and 0.64 between MCI and normal cognition.

**Conclusions:**

In this Chinese population, AD plasma biomarkers vary by age, sex, and *APOE* genotype. Plasma Aβ, t-tau, and NfL differ across the AD clinical spectrum, and plasma NfL appears to be superior to plasma Aβ and t-tau for defining the clinical spectrum.

## INTRODUCTION

The clinical continuum of Alzheimer’s disease (AD) can be divided into three stages: normal cognition, mild cognitive impairment (MCI), and clinical dementia [1]. Amyloid-β (Aβ) plaques, neurofibrillary tangles, and neurodegeneration in the brain are pathophysiological hallmarks of AD [2]. Molecular neuroimaging studies revealed that Aβ in the brain begin to appear up to 20–30 years prior to clinical manifestations of AD [3]. These pathophysiologic changes can be detected either in cerebrospinal fluid (CSF) or in the brain via positron emission tomography (PET) imaging techniques [1]; however, these methods are invasive and relatively expensive. Several blood-based AD biomarkers have in recent years emerged as more accessible, less invasive, and more cost-effective indicators for early detection of MCI and AD [4, 5]. Specifically, plasma Aβ_42_/Aβ_40_ ratio, which correlates with brain amyloid PET load, is considered a reliable biomarker of Aβ pathology in the brain, whereas plasma neurofilament light chain (NfL) and total tau (t-tau) are biomarkers for neurodegeneration [6–8].

Several studies have shown that plasma concentrations of AD biomarkers may vary substantially across ethnoracial groups [9–12]. For example, Mexican Americans had lower plasma Aβ_40_ and NfL, and higher plasma t-tau levels compared to non-Hispanic whites [10, 12]. In addition, genetic background, socioeconomic status, lifestyle factors, and comorbid diseases, which vary across ethnic groups, may influence the racial differences in AD biomarkers [9, 13]. However, the large majority of the previous community-based studies regarding AD plasma biomarkers have been conducted in North America and Europe [12, 14–18]. Exploring the demographic distributions of these plasma biomarkers and their utilities in defining the AD clinical spectrum among the underrepresented ethnical populations (e.g., Chinese population) is highlyrelevant.

The distributions of AD plasma biomarkers across AD clinical spectrum were described in only a few population-based studies. For instance, the Mayo Clinic Study of Aging reported higher plasma t-tau in people with AD than those with normal cognition or MCI, but no difference between normal cognition and MCI [19–21]. The community-based multi-ethnic Health and Aging Brain among Latino Elders study indicated a gradient increase in plasma NfL from normal cognition through MCI to AD, with the area under the receiver operating characteristic (ROC) curve (AUC) for detecting MCI and dementia being 0.55 and 0.70, respectively [12]. However, very few population-based studies have explored simultaneously multiple AD plasma biomarkers for differentiating the AD clinical spectrum. This is important to deepen the understanding of plasma biomarker signatures for AD clinical spectrum. Data from the Washington Heights-Inwood Columbia Aging Project (*n* = 300) showed that plasma Aβ_42_/Aβ_40_ ratio, t-tau, and NfL had poor ability to detect clinical AD (AUC <0.6) [15]. However, the accuracy of AD plasma biomarkers for defining the AD clinical spectrum among Chinese older adults has not yet been evaluated.

Therefore, in this population-based study of rural-dwelling older adults, we aimed to 1) describe the distribution of plasma Aβ, t-tau, and NfL by demographics and apolipoprotein E (*APOE*) genotype; 2) examine the associations of these biomarkers with the clinical spectrum from normal cognition through MCI to clinical AD; and 3) evaluate the performance of these biomarkers to differentiate individuals with normal cognition, MCI, and AD.

## METHODS

### Study participants

**Fig. 1:**
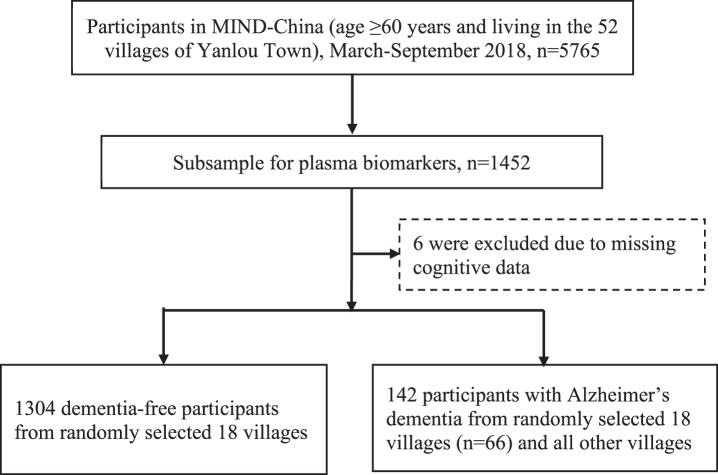
Flowchart of the study participants.

This population-based study used data from a subsample of participants in the baseline assessments of the Multimodal Interventions to Delay Dementia and Disability in Rural China (MIND-China) study, as previously described [22, 23]. In brief, as a participating project in the World-Wide FINGERS Network [24], MIND-China engaged people who were aged ≥60 years and living in the rural communities (52 villages) of Yanlou Town, Yanggu County, western Shandong Province, China. In March-September 2018, 5,765 residents (74.9% of all eligible persons) underwent the baseline examination. Plasma AD biomarkers (i.e., plasma Aβ_40_, Aβ_42_, t-tau, and NfL) were measured in a subsample of 1,304 dementia-free participants who were living in the 18 villages that were randomly selected from the 52 villages plus 142 persons who had blood samples and were diagnosed with AD in all MIND-China participants (of these persons with AD, 66 were from the 18 villages) (Fig. 1). Compared with participants who did not have data on plasma AD biomarkers (*n* = 4,319), those in the plasma biomarker substudy (*n* = 1,446) were slightly younger (mean age 70.2 versus 71.1 years, *p* < 0.001) and more likely to be women (61.0% versus 55.9%, *p* < 0.001), but the two groups did not differ significantly in educational level.

The MIND-China project was approved by the Ethics Committee at Shandong Provincial Hospital affiliated to Shandong University in Jinan, Shandong. Written informed consents were obtained from all participants, or in case of people with severe cognitive impairment, from informants. This study was carried out in accordance with the ethical principles expressed in the Declaration of Helsinki. MIND-China was registered in the Chinese Clinical Trial Registry (Registration No: ChiCTR1800017758).

### Data collection and assessments

Following a structured questionnaire, the trained medical staff collected data through face-to-face interviews, clinical examinations, neuropsychological testing, and laboratory tests, which included sociodemographic features (age, sex, and education), behavioral factors (e.g., smoking and alcohol consumption), metabolic factors (e.g., blood pressure, diabetes, and lipids), health conditions (e.g., coronary heart disease and stroke), use of medications (e.g., antihypertensive agents, blood glucose-lowering drugs, and cholesterol-lowering agents), and *APOE* genotype. Definitions, assessments, and categorizations of covariates are described elsewhere [25, 26] and provided in the Supplementary Material as well.

### Neuropsychological assessments and clinical diagnosis of AD and MCI

Cognitive function was evaluated using a neuropsychological test battery, as previously reported [22, 23]. In brief, we assessed function of the following four cognitive domains: memory was assessed using the Auditory Verbal Learning Test immediate recall, the long-delayed free recall, and the long-delayed recognition; verbal fluency was assessed using the Verbal Fluency Test, including animal, fruit, and vegetable categories; attention was assessed with the Trail Making Test-A and Digit Span Forward test; and executive function was assessed using the Trail Making Test-B and Digit Span Backward test. Each of the raw test scores was standardized into z score using the mean and standard deviation (SD), derived from all participants who were free from dementia. Then, the composite z score for each of the cognitive domains was calculated by averaging the z scores of all the tests for that domain.

The diagnostic procedure of AD and MCI in the MIND-China study has been described elsewhere [22, 23]. In brief, dementia was clinically diagnosed according to the Diagnostic and Statistical Manual of Mental Disorders, Fourth Edition (DSM-IV) criteria [27], following a three-step diagnostic procedure. That is, the trained medical staff first conducted clinical and neurological examinations to assess health-related factors, medical history, and cognitive function following structured questionnaires. Then, neurologists specialized in dementia care reviewed all of the data collected from the initial assessments and made a preliminary judgement for people who were suspected to have dementia. Finally, the neurologists conducted the second in-person interviews with those who were suspected to have dementia or who had insufficient data for making a diagnosis of dementia status, and reassessed their medical history, cognitive status, Chinese version of activity of daily living, and whenever available, brain imaging data. In the case of uncertainty, a senior neurologist was consulted and a consensus diagnosis of dementia was reached. Clinical diagnosis of AD dementia was made according to the National Institute on Aging and Alzheimer’s Association (NIA-AA) criteria for probable AD dementia [28]. MCI was defined according to the Petersen’s criteria [29], where both neuropsychological test scores and a consensus agreement among the examining neurologists were considered, as previously described [23].

### Measurement of plasma biomarkers

After an overnight fast, peripheral blood samples were drawn into ethylene diamine tetraacetic acid (EDTA) citrate vacutainer tubes and centrifuged in a tabletop centrifuge. Plasma samples were then aliquoted and stored at –80°C until retrieved and thawed on ice. Plasma biomarkers were measured using the Single molecule array (Simoa) platform (Quanterix Corp, MA, USA) for Aβ_42_ and Aβ_40_, t-tau (Human Neurology 3-Plex A assay), and NfL (NF-light® advantage Kit) at the laboratory of Wayen Biotechnologies Inc., Shanghai, China. Two quality control plasma samples were run in duplicate on each plate for each analyte. The intra-assay coefficient of variation and the inter-assay coefficient of variation were all below 13.0% for the control sample.

### Statistical analysis

We conducted descriptive statistical analysis to report means (SD) for continuous and frequency (%) for categorical variables. Plasma Aβ_40_ and NfL were natural log-transformed to reduce skewness. Outliers (>5 SDs above the mean of the whole sample) were excluded from the main analysis. We compared characteristics of study participants among normal cognition, MCI, and AD groups using Kruskal-Wallis tests for continuous variables with skewed distribution, and chi-square test for categorical variables. Spearman’s rank order correlation was used to assess correlations between plasma biomarkers (plasma Aβ_42_, Aβ_40_, Aβ_42_/Aβ_40_ ratio, t-tau, and NfL). We used the general linear regression models to examine the associations of demographic factors and *APOE* genotype with plasma biomarkers in the total sample and among dementia-free individuals as previously reported [16]. Multinomial logistic regression analyses were used to estimate the odds ratio (OR) and 95% confidence interval (CI) of plasma biomarkers associated with MCI and AD, in which plasma biomarkers were analyzed as both continuous and categorical (tertiles) variables. We evaluated interactions of plasma biomarkers with age groups (<75 versus ≥75 years), sex, education, or *APOE* *ɛ*4 allele on the likelihood of MCI and AD. Stratifying analysis was then performed when a statistical interaction was detected. We presented the main results from two models: model 1 was adjusted for age, sex, and education, and model 2 was further adjusted for body mass index, smoking, alcohol drinking, estimated glomerular filtration rate (calculated from serum creatinine), hypertension, diabetes, dyslipidemia, coronary heart disease, stroke, and *APOE* genotype. In addition, we used ROC analysis to assess the ability of plasma biomarkers to differentiate between diagnostic groups. Youden index was used to determine optimal sensitivity and specificity. Differences in AUCs were evaluated using Bootstrap test with N = 2000 repetitions [30]. We used Bootstrap test to evaluate to what extent plasma biomarkers alone and in combination with demographic factors and *APOE* genotype could improve the diagnostic accuracy.

We used SAS version 9.4 (SAS Institute Inc., Cary, NC) and R version 4.1.3 for Windows (R Core Team, R Foundation for Statistical Computing, Vienna, Austria. www.R-project.org/) for all the data analyses.

## RESULTS

### Characteristics of the study participants

Out of the 1,446 participants, 402 (27.8%) were defined with MCI and 142 (9.8%) were diagnosed with AD dementia. The mean age of all participants was 70.22 years (SD 5.30), 61.0% were women, and 40.3% were illiterate (Table 1). Compared with normal cognition, participants with MCI or AD were older, less educated, more likely to be women, less likely to smoke and drink alcohol, had lower estimated glomerular filtration rate and plasma Aβ_42_/Aβ_40_ ratio, and higher plasma Aβ_40_ and NfL concentrations (*p* < 0.05) (Table 1). In addition, participants with AD had lower body mass index and higher plasma Aβ_42_ and t-tau than those with normal cognition. Participants with MCI had a higher prevalence of hypertension and stroke than those with normal cognition (*p* < 0.05) (Table 1). The three groups had no significant differences in the proportion of *APOE* *ɛ*4 allele, coronary heart disease, diabetes mellitus, and hypercholesterolemia.

**Table 1:** Characteristics of the study participants by cognitive status (*n* = 1446)

	Total sample	AD cognitive continuum			
Characteristics^a^	(*n* = 1,446)	Normal (*n* = 902)	MCI (*n* = 402)	AD (*n* = 142)	*p*
Age, y	70.22 (5.30)	69.10 (4.29)	70.55 (5.13)^*^	76.35 (7.00)^*†^	<0.001
Women	882 (61.00)	492 (54.55)	274 (68.16)^*^	116 (81.69)^*†^	<0.001
Illiteracy	582 (40.25)	279 (30.93)	194 (48.26)^*^	109 (76.76)^*†^	<0.001
*APOE* *ɛ*4 carrier	227 (16.07)	135 (15.34)	64 (16.33)	28 (19.86)	0.39
Body mass index, kg/m^2^	24.98 (3.59)	25.10 (3.49)	25.14 (3.63)	23.82 (3.96)^*†^	<0.001
Current smoking	274 (18.95)	207 (22.95)	59 (14.68)^*^	8 (5.63)^*†^	<0.001
Current alcohol drinking	397 (27.72)	318 (35.53)	67 (16.92)^*^	12 (8.51)^*†^	<0.001
Stroke	197 (13.62)	107 (11.86)	68 (16.92)^*^	22 (15.49)	0.04
Coronary heart disease	296 (20.47)	180 (19.96)	77 (19.15)	39 (27.46)	0.09
Hypertension	1,002 (69.73)	607 (67.75)	301 (75.25)^*^	94 (66.67)	0.02
Diabetes mellitus	222 (15.35)	143 (15.85)	56 (13.93)	23 (16.20)	0.64
Hypercholesterolemia	234 (16.18)	144 (15.96)	61 (15.17)	29 (20.42)	0.33
eGFR, mL/min/1.73 m^2^	90.71 (18.39)	92.22 (17.38)	89.72 (18.84)^*^	83.99 (21.53)^*†^	<0.001
**Plasma biomarkers**
Aβ_40_, pg/ml	179.21 (46.48)	174.61 (45.62)	183.04 (44.46)^*^	197.58 (52.04)^*†^	<0.001
Aβ_42_, pg/ml	11.97 (3.01)	11.85 (2.91)	12.00 (2.90)	12.67 (3.82)^*^	0.04
Aβ_42_/Aβ_40_ ratio (×100)	6.87 (1.62)	6.99 (1.64)	6.72 (1.56)^*^	6.53 (1.58)^*^	<0.001
Total-tau, pg/ml	2.36 (0.98)	2.32 (0.93)	2.32 (1.01)	2.77 (1.09)^*†^	<0.001
NfL, pg/ml^b^	15.16 (10.00)	13.87 (8.58)	15.68 (10.78)^*^	21.96 (12.94)^*†^	<0.001

MCI, mild cognitive impairment; AD, Alzheimer’s disease; *APOE*, apolipoprotein E gene; eGFR, estimated glomerular filtration rate; Aβ, amyloid-β; NfL, neurofilament light chain. ^a^Number of participants with missing values was 33 for *APOE* genotype, 8 for body mass index, 14 for alcohol drinking, and 9 for hypertension. ^b^Eight outliers with plasma NfL values 5 SDs above the mean of the whole data, as well as one plasma NfL value below the lower limit of quantification were excluded from the analysis (*n* = 1437, of these, 897 with normal cognition, 400 with MCI, and 140 with AD). ^*^p < 0.05 for the comparison with normal cognition. ^†^*p* < 0.05 for the comparison with MCI.

### Correlations between plasma biomarkers

Spearman rank correlation analysis suggested significant correlations of plasma Aβ_40_ with Aβ_42_ (*r* = 0.57), t-tau (*r* = 0.30), and NfL (*r* = 0.27), of plasma Aβ_42_ with t-tau (*r* = 0.15) and NfL (*r* = 0.20), of plasma Aβ_42_/Aβ_40_ ratio with t-tau (*r* = –0.14) and NfL (*r* = –0.07), and of plasma t-tau with NfL (*r* = 0.20) (for all correlation coefficients, *p* < 0.001) (Supplementary Figure 1).

### Associations of plasma biomarkers with demographic factors and APOE *ɛ*4 allele

**Fig. 2:**
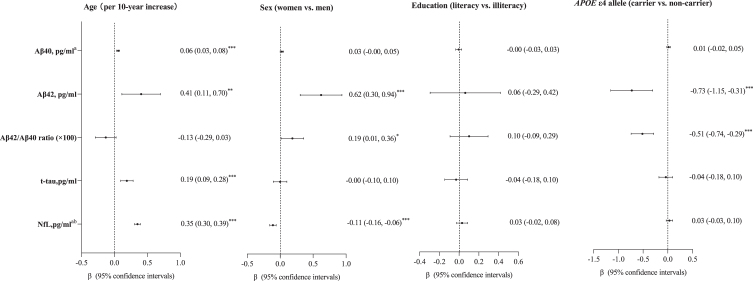
Associations of age, sex, education, and *APOE* *ɛ*4 allele with plasma biomarkers. Aβ, amyloid-β; t-tau, total-tau; NfL, neurofilament light chain; *APOE*, apolipoprotein E gene. ^a^These data were natural log-transformed to normalize the distributions (*n* = 1,446, 402 with mild cognitive impairment, 142 with Alzheimer’s disease). ^b^Eight outliers with plasma NfL values >5 SDs above the mean of the whole data, as well as one plasma NfL value below the lower limit of quantification was excluded from the main analysis (*n* = 1437, 400 with mild cognitive impairment and 140 with Alzheimer’s disease). ^*^p < 0.05; ^**^p < 0.01; ^***^p < 0.001. Model was adjusted for age and sex wherever appropriate.

Plasma Aβ_40_, Aβ_42_, t-tau, and NfL, but not Aβ_42_/Aβ_40_ ratio, increased with advanced age (Fig. 2). Furthermore, after controlling for age, plasma Aβ_42_, and the Aβ_42_/Aβ_40_ ratio were higher in women than in men, whereas plasma NfL was lower in women than in men (for all sex differences, *p* < 0.05) (Fig. 2). In addition, *APOE* *ɛ*4 allele was significantly associated with lower plasma Aβ_42_ and Aβ_42_/Aβ_40_ ratio in the age- and sex-adjusted model (Fig. 2). Education was not significantly associated with any of the examined plasma biomarkers. All these associations remained largely the same after adjustment for demographic factors, *APOE* genotype, lifestyles, metabolic factors, and clinical factors (Supplementary Figure 2).

Among individuals free of dementia, the associations of plasma Aβ_40_, Aβ_42_, and NfL with increased age remained statistically significant, but the correlation between plasma t-tau and advanced age became statistically non-significant (Supplementary Figure 3).

### Associations of plasma biomarkers with the cognitive continuum

**Fig. 3:**
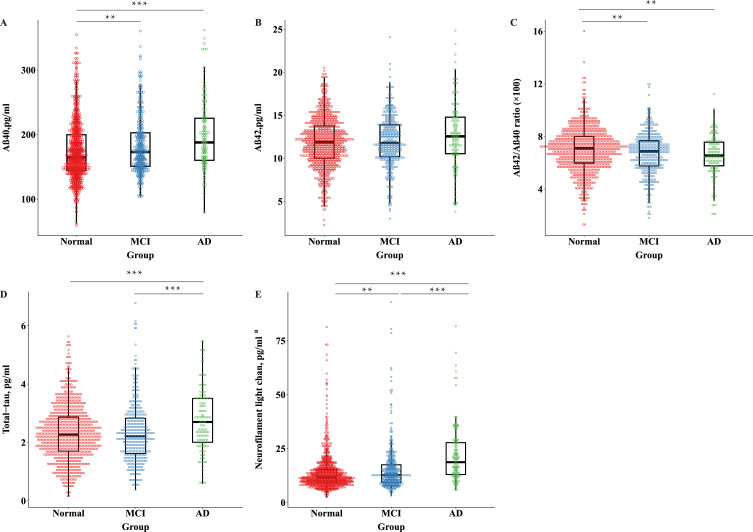
Distribution of AD plasma biomarkers across groups of people with normal cognition, mild cognitive impairment, and Alzheimer’s disease. MCI, mild cognitive impairment; AD, Alzheimer’s disease; Aβ, amyloid-β; NfL, neurofilament light chain. ^a^Eight outliers with plasma NfL values >5 SDs above the mean of the whole data, as well as one below the lower limit of quantification were excluded from the main analysis (*n* = 1437, 400 with mild cognitive impairment, 140 with Alzheimer’s disease). ^*^p < 0.05; ^**^p < 0.01; ^***^p < 0.001. All *p* values are derived from the general linear model, adjusting for age, sex, and education.

Participants with AD (versus normal cognition group) had higher plasma Aβ_40_, t-tau, and NfL, and lower Aβ_42_/Aβ_40_ ratio (demographic-adjusted *p* < 0.01) (Fig. 3). Participants with AD dementia showed higher plasma t-tau and NfL than those with MCI (demographic-adjusted *p* < 0.01). In addition, participants with MCI showed higher plasma Aβ_40_ and NfL, and a lower Aβ_42_/Aβ_40_ ratio than those with normal cognition (demographic-adjusted *p* < 0.01) (Fig. 3). Same results were obtained in fully-adjusted models.

Similarly, higher plasma Aβ_40_ and NfL, and a lower Aβ_42_/Aβ_40_ ratio were significantly related to a higher likelihood of MCI after controlling for demographic factors (Table 2, Model 1). In addition, higher plasma Aβ_40_, t-tau, and NfL, and a lower Aβ_42_/Aβ_40_ ratio were significantly associated with a higher likelihood of AD after adjusting for demographic factors (Table 2, Model 1). When plasma Aβ, t-tau, and NfL were analyzed as tertiles, similar patterns of associations with MCI and AD were obtained (Table 2, Model 1). All these associations remained statistically significant when further controlling for *APOE* genotype, lifestyle, metabolic factors, and clinical factors, except that the association between medium tertile of Aβ_40_ and an increased likelihood of AD was attenuated and became statistically non-significant (Table 2, Model 2).

**Table 2:** Odds ratios and 95% confidence intervals of mild cognitive impairment and Alzheimer’s disease with plasma biomarkers from multinomial logistic regression models (*n* = 1,446)

Plasma biomarkers	Mild cognitive impairment (*n* = 402)		Alzheimer’s disease (*n* = 142)	
	Model 1^†^	Model 2^†^	Model 1^†^	Model 2^†^
**Aβ_**40**_, pg/ml^**a**^**
Continues	2.16 (1.32–3.53)^**^	1.99 (1.19–3.33)^**^	4.50 (1.94–10.45)^***^	3.27 (1.33–8.00)^**^
Categorical (tertiles)
Lower	1.00 (reference)	1.00 (reference)	1.00 (reference)	1.00 (reference)
Medium	1.52 (1.13–2.05)^**^	1.56 (1.15–2.12)^**^	1.75 (1.00, 3.05)^*^	1.60 (0.90–2.83)
Upper	1.52 (1.12–2.05)^**^	1.44 (1.05–1.97)^*^	2.28 (1.33–3.90)^**^	1.84 (1.05–3.23)^*^
*p* for trend	<0.01	0.02	<0.01	0.03
**Aβ_**42**_, pg/ml**
Continues	1.01 (0.97–1.05)	1.00 (0.96–1.04)	1.05 (0.98–1.12)	1.02 (0.95–1.10)
Categorical (tertiles)
Lower	1.00 (reference)	1.00 (reference)	1.00 (reference)	1.00 (reference)
Medium	1.14 (0.84–1.53)	1.10 (0.81–1.49)	1.05 (0.62–1.78)	0.91 (0.53–1.57)
Upper	1.09 (0.81–1.47)	1.03 (0.75–1.40)	1.12 (0.68–1.86)	0.91 (0.53–1.55)
*p* for trend	0.59	0.88	0.64	0.74
**Aβ_**42**_/Aβ_**40**_ ratio (×100)**
Continues	0.89 (0.83–0.96)^**^	0.90 (0.83–0.97)^**^	0.84 (0.74–0.96)^**^	0.85 (0.74–0.97)^*^
Categorical (tertiles)
Lower	1.00 (reference)	1.00 (reference)	1.00 (reference)	1.00 (reference)
Medium	0.99 (0.74–1.32)	1.04 (0.77–1.40)	1.08 (0.66–1.76)	1.05 (0.63–1.75)
Upper	0.64 (0.47–0.86)^**^	0.65 (0.48–0.88)^**^	0.63 (0.37–1.05)	0.65 (0.38–1.11)
*p* for trend	<0.01	<0.01	0.08	0.11
**T-tau, pg/ml**
Continues	0.99 (0.88–1.13)	0.99 (0.87–1.13)	1.37 (1.12–1.67)^**^	1.39 (1.13–1.70)^**^
Categorical (tertiles)
Lower	1.00 (reference)	1.00 (reference)	1.00 (reference)	1.00 (reference)
Medium	1.00 (0.75–1.33)	1.02 (0.76–1.37)	1.18 (0.68–2.05)	1.30 (0.74–2.30)
Upper	0.82 (0.61–1.11)	0.81 (0.60–1.11)	1.77 (1.06–2.95)^*^	1.89 (1.11–3.22)^*^
*p* for trend	0.21	0.20	0.02	0.02
**NfL , pg/ml ^**ab**^**
Continues	1.51 (1.15–1.97)^**^	1.46 (1.10–1.93)^**^	3.42 (2.25–5.21)^***^	2.96 (1.90–4.62)^***^
Categorical (tertiles)
Lower	1.00 (reference)	1.00 (reference)	1.00 (reference)	1.00 (reference)
Medium	0.96 (0.71–1.30)	0.94 (0.69–1.28)	1.34 (0.71–2.53)	1.33 (0.70–2.54)
Upper	1.42 (1.04–1.96)^*^	1.42 (1.02–1.99)^*^	3.27 (1.77–6.02)^***^	2.85 (1.50–5.42)^**^
*p* for trend	0.03	0.04	<0.001	<0.001

Aβ, amyloid-β; t-tau, total tau; NfL, neurofilament light chain. ^a^These data were natural log-transformed to normalize the distributions. ^b^Eight outliers with plasma NfL values >5 SDs above the mean of the whole data, as well as one below the lower limit of quantification were excluded from the main analysis (*n* = 1437, 400 with mild cognitive impairment, 140 with Alzheimer’s disease). ^*^p < 0.05; ^**^p < 0.01; ^***^p < 0.001. ^†^Participants with normal cognition (*n* = 902) were used as a referent group in the multinomial logistic regression analysis. Model 1 was adjusted for age, sex, and education; and in model 2, additional adjustment was made for *APOE* genotype, behavioral, metabolic, and clinical factors.

In addition, we detected statistical interactions of plasma Aβ_42_/Aβ_40_ ratio with *APOE* *ɛ*4 allele on the likelihood of AD (p for interaction = 0.02), such that a higher Aβ_42_/Aβ_40_ ratio was significantly associated with a lower odds ratio of AD among *APOE* *ɛ*4 allele non-carriers (multivariable-adjusted OR = 0.79; 95% CI 0.68–0.91), but not among *APOE* *ɛ*4 allele carriers (1.23; 0.87–1.74).

### Performance of plasma biomarkers in discriminating the cognitive continuum

**Fig. 4:**
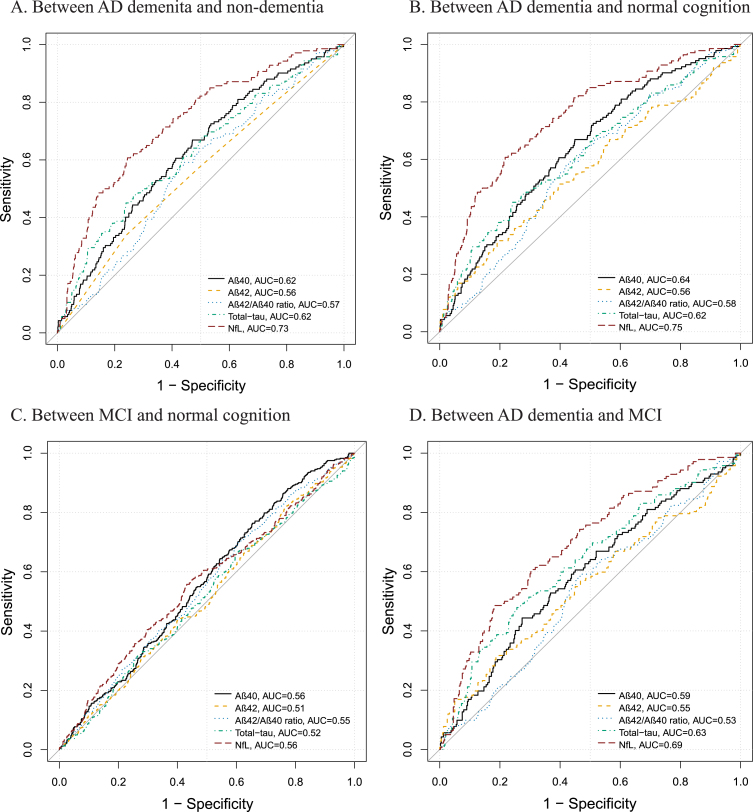
Discriminative performance of plasma biomarkers across diagnostic groups. Receiver-operating characteristics curves displaying the performance of plasma Aβ_40_, Aβ_42_, Aβ_42_/Aβ_40_ ratio, total-tau, and NfL to distinguish (A) between AD dementia and non-dementia; (B) between AD dementia and normal cognition, (C) between MCI and normal cognition, and (D) between AD dementia and MCI. MCI, mild cognitive impairment; AD, Alzheimer’s disease; Aβ, amyloid-β; NfL, neurofilament light chain; AUC, areas under the receiver operating characteristics curve.

Of all the examined plasma biomarkers, plasma NfL showed the highest AUC value for differentiating between AD and the combined non-dementia groups (i.e., MCI plus normal cognition) (AUC = 0.73; 95% CI 0.69–0.77; Fig. 4A), between AD and normal cognition (0.75; 0.70–0.79; Fig. 4B), and between AD and MCI (0.69; 0.64–0.74; Fig. 4D). Plasma t-tau showed similar AUC to that of plasma NfL for discriminating AD from MCI (0.63; 0.58–0.68; Fig. 4D) (p for bootstrap test >0.05). All the examined plasma biomarkers performed poorly in differentiating between MCI and normal cognition (Fig. 4C).

Adding age, sex, education, and *APOE* *ɛ*4 allele to plasma NfL in the models significantly improved the accuracy for discriminating AD from non-dementia (AUC = 0.85:95% CI 0.81–0.88), normal cognition (0.87; 0.84–0.91), and MCI (0.79; 0.74–0.83) (compared with plasma NfL alone, all *p* for bootstrap test <0.001), but not for differentiating between MCI and normal cognition (*p* for bootstrap test >0.05) (Supplementary Table 1).

## DISCUSSION

In this population-based study of rural-dwelling older adults in China, we characterized the plasma biomarkers of amyloid and neurodegeneration associated with the cognitive continuum from normal cognition through MCI to AD and further evaluated diagnostic performance of the plasma biomarkers. The main findings can be summarized as follows: 1) plasma Aβ, t-tau, and NfL varied by age, sex, or *APOE* genotype such that older age was associated with higher plasma Aβ_40_, Aβ_42_, t-tau, and NfL; that women had higher plasma Aβ_42_ but lower NfL than men; and that *APOE* *ɛ*4 carriers had lower plasma Aβ_42_ and Aβ_42_/Aβ_40_ ratio than non-carriers; 2) plasma Aβ_40_, t-tau, and NfL concentrations increased while the Aβ_42_/Aβ_40_ ratio decreased from normal cognition through MCI to AD; and 3) plasma NfL outperformed all other examined biomarkers in distinguishing people with AD dementia from those with MCI or with normal cognition.

The increase in plasma Aβ_40_, Aβ_42_, t-tau, and NfL with advanced age was consistent with the reports from the Mayo Clinic Study of Aging [14] and the Rotterdam Study [16]. From the perspective of neuropathology, the age-dependent increase in plasma Aβ might be due to the increased Aβ production and decreased Aβ clearance with advanced age [31]. The age-dependent increase in neurodegeneration (e.g., NfL) might partly contribute to the accumulative subclinical comorbid pathologies in old age such as cerebrovascular lesions and neuroinflammation as well as injuries of neuron and axon[32, 33].

Previously, the population-based studies and the meta-analysis revealed higher plasma and CSF NfL concentrations in men than in women [12, 34], which was consistent with our study, although some other studies did not find such a sex difference [14, 15, 17]. In addition, two population-based studies reported no sex difference of plasma t-tau [15, 18], which was in line with our observation, but two other population-based studies did report higher plasma t-tau in women than in men [14, 17]. The differences in ethnicity/race, comorbid diseases or lifestyle of the study participants may partly contribute to discrepancies in findings across studies. The sex differences in plasma Aβ and neurodegenerative biomarkers warrant further investigation in various ethnic populations.

In addition, we found that *APOE* *ɛ*4 allele was associated with lower plasma Aβ_42_ and Aβ_42_/Aβ_40_ ratio but not with plasma Aβ_40_, consistent with the findings from the Rotterdam study [35] and a genome-wide association study [36]. The Aβ_42_, the primary component of amyloid plaque, is more likely to aggregate than Aβ_40_. In addition, the apoE4 protein, coded by the *ɛ*4 allele variant of the *APOE* gene, is known to influence the Aβ aggregation or clearance process rather than the process of Aβ peptide production [36].

We found that across the AD clinical spectrum, plasma Aβ_40_ increased and the Aβ_42_/Aβ_40_ ratio decreased, whereas there was no difference in plasma Aβ_42_. This was consistent with the Atherosclerosis Risk in Communities Neurocognitive study, which reported that higher plasma Aβ_40_ and lower plasma Aβ_42_/Aβ_40_ ratio in midlife and late-life were associated with MCI and dementia and that lower plasma Aβ_42_ at midlife, but not late-life, was related to MCI or dementia [37]. However, previous cross-sectional studies regarding the association of plasma Aβ with dementia have yielded mixed results because both elevated and decreased plasma Aβ_42_ levels were reported in people with cognitive impairment [38, 39]. In addition, plasma Aβ_42_ levels might increase in the pre-pathological stage of AD and then decrease with progression of the disease [40], and as a result, plasma Aβ_42_ levels may decline into normal ranges in the clinical phase. Thus, plasma Aβ measured at different stages of AD may partly interpret the discrepancies. Furthermore, we found that plasma t-tau concentration was higher in people with AD than those with MCI or normal cognition, which was in line with the report from the Mayo Clinic Study of Aging [20, 21]. This suggests that plasma t-tau could be a biomarker at the relatively late stages of the AD clinical continuum [16, 41]. Our data were also in agreement with a community-based multi-ethnic study in US [12], which showed that plasma NfL levels were increased across the AD clinical spectrum.

A lower plasma Aβ_42_/Aβ_40_ ratio might reflect more Aβ aggregation in the brain. Indeed, recent cohort studies showed that plasma Aβ_42_/Aβ_40_ ratio could be used to identify people with abnormal CSF or PET amyloid status [42] and that a low plasma Aβ_42_/Aβ_40_ ratio was associated with a high load of cerebral AD pathology [43]. However, our data suggested that plasma Aβ_42_/Aβ_40_ ratio was not sensitive in differentiating cognitive phenotypes in old age. This may be related to the very low effect size (fold change) in amyloid positive versus negative individuals, resulting in a low robustness for this blood biomarker [44]. Indeed, plasma Aβ levels may reflect only to some extent the Aβ aggregation in the brain due to peripheral Aβ generation, degradation by circulating enzymes, and metabolism in the liver [45]. In addition, the available Aβ assay was specific for x-40 and x-42 rather than 1–40 and 1–42, which was less disease-specific [46], and the head-to-head study showed that certain mass spectroscopy-based methods performed better than immunoassays for plasma Aβ_42_/Aβ_40_ ratio when detecting Aβ pathology in the brain [47]. Plasma t-tau is considered a biomarker of neuronal damage and neurodegeneration. However, our study showed that plasma t-tau was elevated only in the late stage of the AD clinical spectrum and the diagnostic performance was poor for plasma t-tau to discriminate AD from non-dementia. This finding is in agreement with data from the Alzheimer’s Disease Neuroimaging Initiative (ADNI) study, showing only a minor increase in plasma t-tau in people with AD dementia compared with healthy controls [48], probably because current assay for plasma t-tau captures both brain-derived and peripherally produced tau protein [49]. Plasma NfL was increased in several neurodegenerative disorders (e.g., frontotemporal dementia, AD, and corticobasal syndrome) [4]. Plasma NfL in combination with demographic features and *APOE* genotype appeared to be powerful in differentiating AD from normal cognition, which is in line with the reports from the ADNI study [50] and a community-based study in US [51].

In this population-based study, we characterized plasma amyloid and neurodegenerative biomarkers across the AD clinical spectrum and assessed their accuracy for differentiating between normal cognition, MCI, and AD among rural-dwelling older adults in China, a sociodemographic group that has been largely ignored in Alzheimer research. Furthermore, we used the state-of-the-art Simoa platform to measure plasma biomarkers. However, our study also has limitations. First, we did not have data of highly sensitive and specific plasma biomarkers for tau pathology (e.g., phosphorylated tau181, tau217, and tau231). Furthermore, we did not have data on biomarkers related to amyloid, tau, and neurodegeneration (ATN) in central nervous system, which are more reliable than peripheral AD-related biomarkers to define pathological features of AD. Finally, our study sample was derived only from only one rural area in China, which should be kept in mind when generalizing the study findings to different populations.

In conclusion, plasma amyloid, t-tau, and NfL concentrations vary with age, sex, and *APOE* genotype and across the AD clinical spectrum. Notably, plasma NfL increased across the continuum from normal cognition to prodromal and clinical AD dementia and could be a valuable biomarker for detecting AD in older adults. Further longitudinal studies are required to evaluate the trajectory of these plasma biomarkers in defining the AD cognitive continuum and their prognostic ability in predicting dynamic evolution of cognitive traits.

## Supplementary Material

Supplementary Material

## Data Availability

The datasets used and/or analyzed during the current study are available from the corresponding author upon reasonable request and approval by the Steering Committee of MIND-China at the Department of Neurology, Shandong Provincial Hospital.
