# Supplementary material for: Plasma Amyloid-β, Total Tau, and Neurofilament Light Chain Across the Alzheimer’s Disease Clinical Spectrum: A Population-Based Study
Source: J Alzheimers Dis. 2023 Nov 7;96(2):845–58. doi: 10.3233/JAD-230932 (PMC10657676; doi:10.3233/JAD-230932)
Supplement: Supplementary Material [file jad-96-jad230932-s001.pdf]

Supplementary Material

Plasma Amyloid-β, Total Tau, and Neurofilament Light Chain Across the Alzheimer's Disease Clinical Spectrum: A Population-Based Study

Supplementary Material 1. Definitions, assessments, and categorizations of covariates

The trained staff collected data through face-to-face interviews, clinical examinations, and laboratory tests, following a structured questionnaire, which included data on sociodemographic features (age, sex, and education), behavioral factors (e.g., smoking and alcohol consumption), metabolic factors (e.g., blood pressure, diabetes, lipids, creatinine, weight, and height), health conditions (e.g., coronary heart disease and stroke), use of medications (e.g., antihypertensive agents, blood glucose-lowering drugs, and cholesterol-lowering agents), and apolipoprotein E (*APOE*) genotype.

All medications were classified and coded according to the Anatomical Therapeutic Chemical (ATC) Classification System [1]. Educational level was dichotomized into illiteracy (no formal schooling education) and literacy. *APOE* genotype was performed using multiple-polymerase chain reaction (iGeneTech Bioscience Co., Ltd., Beijing, China) and was dichotomized into carriers versus non-carriers of the ε4 allele. Smoking and alcohol consumption were dichotomized into never or former, and current smoking or alcohol intake. Weight and height were measured light clothing without shoes. Body mass index (BMI) was calculated as weight (kg) divided by height (m^2). Peripheral blood samples were taken after an overnight fast. Blood glucose, lipids and creatine were measured at the Yanlou Town Hospital laboratory following standardized protocol. Estimated glomerular filtration rate (eGFR) was calculated using Chinese improved Modification of Diet in Renal Disease equation [2]. Arterial blood pressure was measured on the right arm using an electronic blood pressure monitor (Omron HEM-7127J; Omron Corporation, Kyoto, Japan) after a 5-min rest. Hypertension was defined as blood pressure ≥140/90 mmHg or current use of antihypertensive medication (ATC codes C02, C03, and C07-C09). Diabetes mellitus was defined as having a self-reported history of diabetes, fasting blood glucose ≥7.0 mmol/L or use of glucose-lowering drugs or insulin injection (ATC code A10). High cholesterol was defined as total cholesterol ≥6.2 mmol/L or use of cholesterol-lowering drugs (ATC codes C10). Coronary heart disease (e.g., angina pectoris, myocardial

infarction, and coronary intervention) was ascertained via self-reported history of physician diagnosis or electrocardiogram examination. History of stroke was determined via self-report or the judgment by a neurologist or physician via clinical and neurological examination.

REFERENCES

[1] Cong L, Ren Y, Hou T, Han X, Dong Y, Wang Y, Zhang Q, Liu R, Xu S, Wang L, Du Y, Qiu C (2020) Use of cardiovascular drugs for primary and secondary prevention of cardiovascular disease among rural-dwelling older Chinese adults. *Front Pharmacol* **11**, 608136.

[2] Zhang L, Wang F, Wang L, Wang W, Liu B, Liu J, Chen M, He Q, Liao Y, Yu X, Chen N, Zhang JE, Hu Z, Liu F, Hong D, Ma L, Liu H, Zhou X, Chen J, Pan L, Chen W, Wang W, Li X, Wang H (2012) Prevalence of chronic kidney disease in China: a cross-sectional survey. *Lancet* **379**, 815-822.

Supplementary Table 1. Diagnostic accuracy of plasma biomarkers between normal cognition, mild cognitive impairment, and Alzheimer's disease

Test	n per group	AUC (95% CI)	Sensitivity	Specificity
**AD versus Non-dementia**				
$A\beta_{40}$, pg/ml	142 versus 1304	0.62 (0.58-0.67)	0.67	0.53
$A\beta_{42}$, pg/ml	142 versus 1304	0.56 (0.51-0.61)	0.32	0.80
$A\beta_{42}/A\beta_{40}$ ratio (×100)	142 versus 1304	0.57 (0.52-0.61)	0.56	0.59
Total-tau, pg/ml	142 versus 1304	0.62 (0.57-0.67)	0.45	0.76
NfL, pg/ml	140 versus 1297	0.73 (0.69-0.77)	0.61	0.76
Base model[a]	141 versus 1272	0.83 (0.79-0.87)	0.68	0.84
Base model+$A\beta_{40}$	141 versus 1272	0.84 (0.80-0.87)	0.70	0.85
Base model+$A\beta_{42}$	141 versus 1272	0.83 (0.79-0.87)	0.69	0.83
Base model+$A\beta_{42}/A\beta_{40}$ ratio	141 versus 1272	0.83 (0.79-0.87)	0.70	0.84
Base model+Total-tau	141 versus 1272	0.84 (0.80-0.88)	0.72	0.84
Base model+NfL	139 versus 1265	0.85 (0.81-0.88)	0.74	0.82
**AD versus Normal cognition**				
$A\beta_{40}$, pg/ml	142 versus 902	0.64 (0.59-0.69)	0.67	0.55
$A\beta_{42}$, pg/ml	142 versus 902	0.56 (0.51-0.62)	0.32	0.80
$A\beta_{42}/A\beta_{40}$ ratio (×100)	142 versus 902	0.58 (0.53-0.63)	0.52	0.64
Total-tau, pg/ml	142 versus 902	0.62 (0.57-0.67)	0.45	0.76
NfL, pg/ml	140 versus 897	0.75 (0.70-0.79)	0.61	0.78
Base model[a]	141 versus 880	0.86 (0.82-0.89)	0.68	0.88
Base model+$A\beta_{40}$	141 versus 880	0.86 (0.83-0.90)	0.72	0.88
Base model+$A\beta_{42}$	141 versus 880	0.86 (0.82-0.89)	0.69	0.87
Base model+$A\beta_{42}/A\beta_{40}$ ratio	141 versus 880	0.86 (0.82-0.90)	0.70	0.89
Base model+Total-tau	141 versus 880	0.86 (0.83-0.90)	0.75	0.86
Base model+NfL	139 versus 875	0.87 (0.84-0.91)	0.74	0.87
**MCI versus Cognitive unimpaired**				
$A\beta_{40}$, pg/ml	402 versus 902	0.56 (0.53-0.59)	0.76	0.35
$A\beta_{42}$, pg/ml	402 versus 902	0.51 (0.48-0.55)	0.82	0.23
$A\beta_{42}/A\beta_{40}$ ratio (×100)	402 versus 902	0.55 (0.52-0.58)	0.40	0.70
Total-tau, pg/ml	402 versus 902	0.52 (0.48-0.55)	0.40	0.66
NfL, pg/ml	400 versus 897	0.56 (0.52-0.59)	0.56	0.57
Base model[a]	392 versus 880	0.63 (0.60-0.67)	0.54	0.66
Base model+$A\beta_{40}$	392 versus 880	0.64 (0.60-0.67)	0.42	0.79
Base model+$A\beta_{42}$	392 versus 880	0.63 (0.60-0.67)	0.57	0.64
Base model+$A\beta_{42}/A\beta_{40}$ ratio	392 versus 880	0.64 (0.61-0.67)	0.53	0.70
Base model+Total-tau	392 versus 880	0.63 (0.60-0.67)	0.56	0.64
Base model+NfL	390 versus 875	0.64 (0.60-0.67)	0.40	0.82
**MCI versus AD**				
$A\beta_{40}$, pg/ml	402 versus 142	0.59 (0.54-0.65)	0.44	0.73
$A\beta_{42}$, pg/ml	402 versus 142	0.55 (0.49-0.61)	0.31	0.81
$A\beta_{42}/A\beta_{40}$ ratio (×100)	402 versus 142	0.53 (0.48-0.59)	0.55	0.58
Total-tau, pg/ml	402 versus 142	0.63 (0.58-0.68)	0.48	0.74
NfL, pg/ml	400 versus 140	0.69 (0.64-0.74)	0.49	0.82
Base model[a]	390 versus 141	0.77 (0.72-0.82)	0.67	0.76

Base model+Aβ_{40}	390 versus 141	0.77 (0.73-0.82)	0.70	0.74
Base model+Aβ_{42}	390 versus 141	0.77 (0.72-0.82)	0.56	0.86
Base model+Aβ_{42}/Aβ_{40} ratio	390 versus 141	0.77 (0.72-0.82)	0.67	0.78
Base model+Total-tau	390 versus 141	0.79 (0.74-0.83)	0.75	0.74
Base model+NfL	390 versus 139	0.79 (0.74-0.83)	0.75	0.71

AUC, areas under the receiver operating characteristics curve; CI, confidence interval; AD, Alzheimer's disease; MCI, mild cognitive impairment; Aβ, amyloid-β; NfL, neurofilament light chain; *APOE*, apolipoprotein E gene.

[a] Base model includes age, sex, education, and *APOE* genotype.

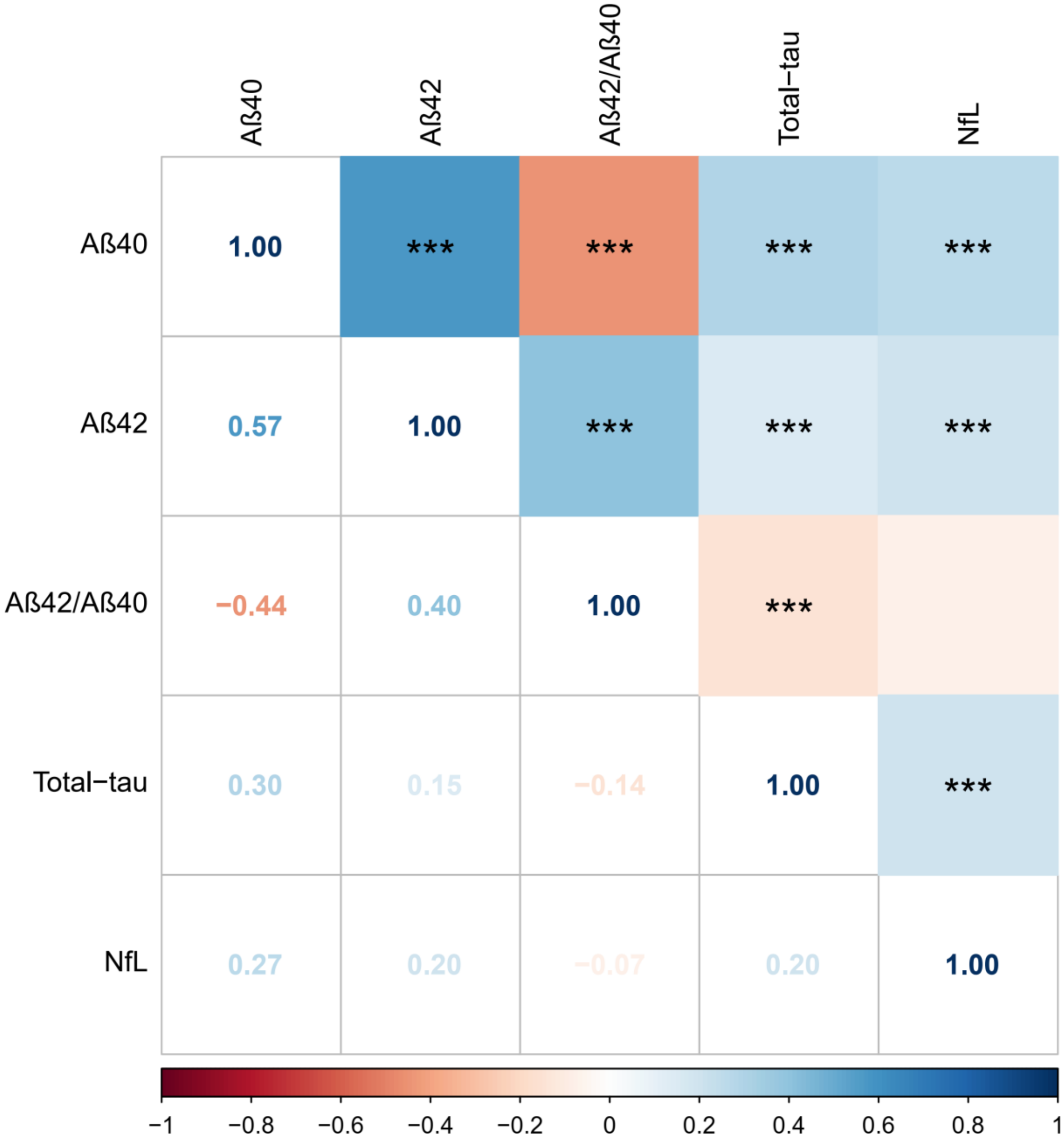

Supplementary Figure 1. Spearman rank correlations between plasma biomarkers.
The figure shows the Spearman rho coefficients. Positive correlations (r>0) were exhibited in blue, and negative correlations (r<0) were exhibited in red in the heatmap figure. Aβ, amyloid beta; NfL, neurofilament light chain. Eight outliers with plasma NfL values >5 SDs above the mean of the whole data, as well as one plasma NfL value below the lower limit of quantification was excluded from the main analysis (n=1,437, 897 with normal cognition, 400 with mild cognitive impairment, and 140 with Alzheimer's disease). ***p<0.001.

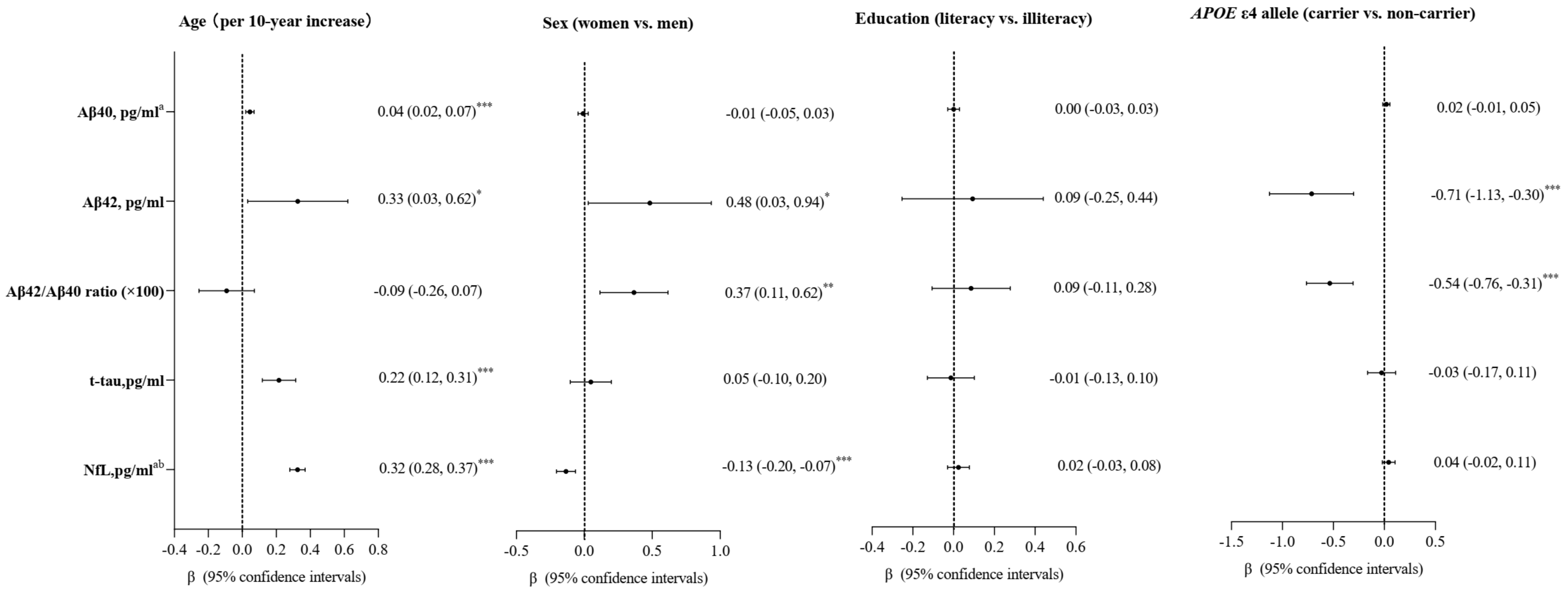

Supplementary Figure 2 Associations of age, sex, education, and *APOE* ε4 allele with plasma biomarkers.
Aβ, amyloid-β; t-tau, total-tau; NfL, neurofilament light chain; *APOE*, apolipoprotein E gene.
[a] These data were natural log-transformed to normalize the distributions (n=1446, 402 with mild cognitive impairment, 142 with Alzheimer's disease).
[b] Eight outliers with plasma NfL values >5 SDs above the mean of the whole data, as well as one plasma NfL value below the lower limit of quantification was excluded from the main analysis (n=1437, 400 with mild cognitive impairment and 140 with Alzheimer's disease).
*p<0.05; **p<0.01; ***p<0.001.
Model was adjusted for age, sex, education, *APOE* genotype, behavioral, metabolic, and clinical factors.

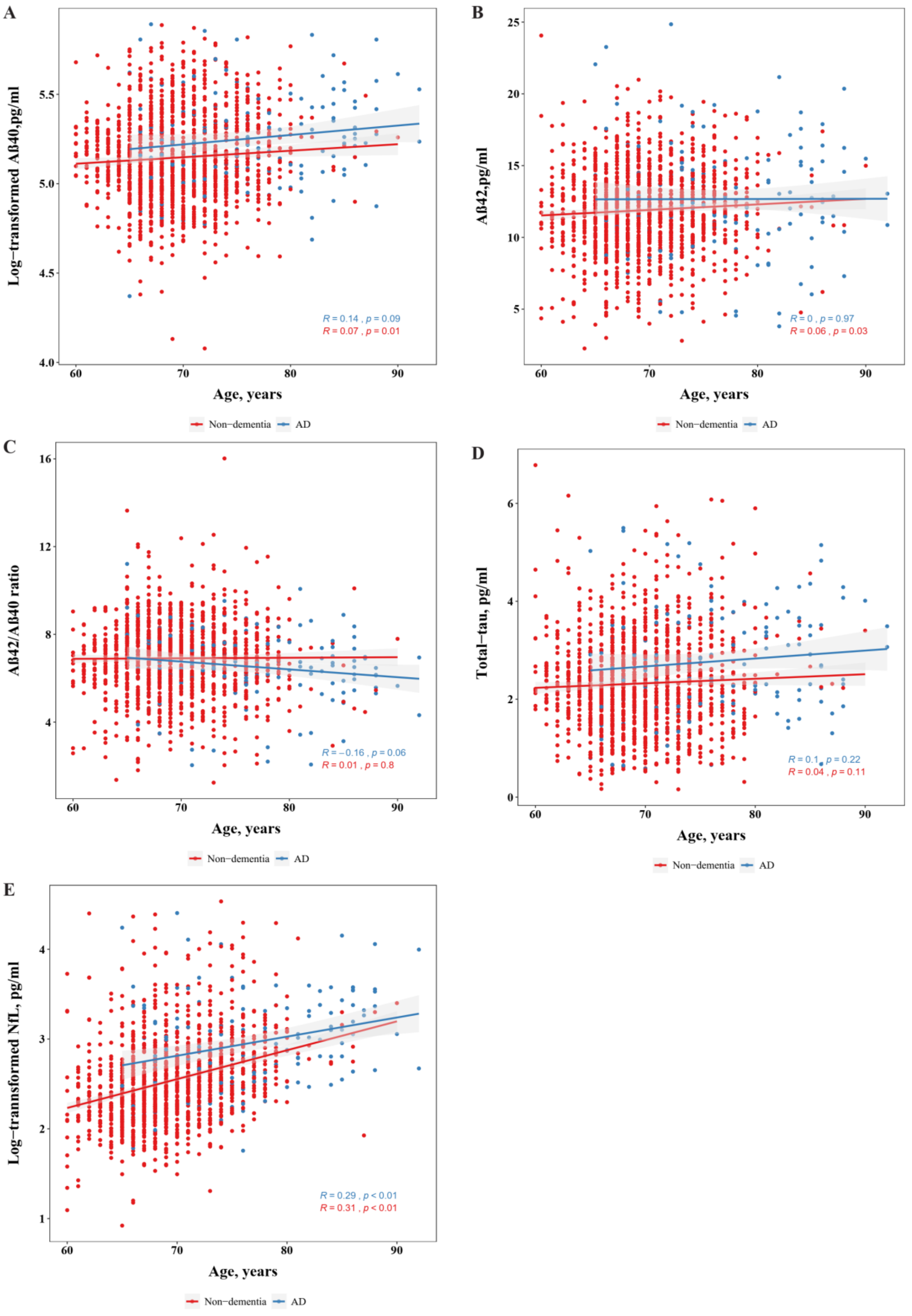

A
Log−transformed Aβ40,pg/ml
Age, years
R = 0.14 , p = 0.09
R = 0.07 , p = 0.01
Non−dementia
AD
B
Aβ42,pg/ml
Age, years
R = 0 , p = 0.97
R = 0.06 , p = 0.03
Non−dementia
AD
C
Aβ42/Aβ40 ratio
Age, years
R = −0.16 , p = 0.06
R = 0.01 , p = 0.8
Non−dementia
AD
D
Total−tau, pg/ml
Age, years
R = 0.1 , p = 0.22
R = 0.04 , p = 0.11
Non−dementia
AD
E
Log−transformed NfL, pg/ml
Age, years
R = 0.29 , p < 0.01
R = 0.31 , p < 0.01
Non−dementia
AD

Supplementary Figure 3. Correlations of plasma biomarkers with age by cognitive status.
The figure shows the Pearson rho coefficients of plasma Aβ_{40} (A), Aβ_{42} (B), Aβ_{40}/Aβ_{42} ratio (C), t-tau (D), and NfL (E) with age. Aβ, amyloid beta; T-tau, total tau; NfL, neurofilament light chain. Eight outliers with plasma NfL values 5 SDs above the mean of the whole data, as well as one plasma NfL value below the lower limit of quantification was excluded from the main analysis (n=1437, 897 with normal cognition, 400 with mild cognitive impairment and 140 with Alzheimer's disease).
